# ATP Release from Dying Autophagic Cells and Their Phagocytosis Are Crucial for Inflammasome Activation in Macrophages

**DOI:** 10.1371/journal.pone.0040069

**Published:** 2012-06-29

**Authors:** Gizem Ayna, Dmitri V. Krysko, Agnieszka Kaczmarek, Goran Petrovski, Peter Vandenabeele, László Fésüs

**Affiliations:** 1 Department of Biochemistry and Molecular Biology, Medical and Health Science Center, University of Debrecen, Debrecen, Hungary; 2 Molecular Signalling and Cell Death Unit, Department for Molecular Biomedical Research, VIB, Ghent, Belgium; 3 Department of Biomedical Molecular Biology, Ghent University, Ghent, Belgium; 4 The Apoptosis and Genomics Research Group of the Hungarian Academy of Sciences; Medical and Health Science Center, University of Debrecen, Debrecen, Hungary; University Paris Sud, France

## Abstract

Pathogen-activated and damage-associated molecular patterns activate the inflammasome in macrophages. We report that mouse macrophages release IL-1β while co-incubated with pro-B (Ba/F3) cells dying, as a result of IL-3 withdrawal, by apoptosis with autophagy, but not when they are co-incubated with living, apoptotic, necrotic or necrostatin-1 treated cells. NALP3-deficient macrophages display reduced IL-1β secretion, which is also inhibited in macrophages deficient in caspase-1 or pre-treated with its inhibitor. This finding demonstrates that the inflammasome is activated during phagocytosis of dying autophagic cells. We show that activation of NALP3 depends on phagocytosis of dying cells, ATP release through pannexin-1 channels of dying autophagic cells, P_2_X_7_ purinergic receptor activation, and on consequent potassium efflux. Dying autophagic Ba/F3 cells injected intraperitoneally in mice recruit neutrophils and thereby induce acute inflammation. These findings demonstrate that NALP3 performs key upstream functions in inflammasome activation in mouse macrophages engulfing dying autophagic cells, and that these functions lead to pro-inflammatory responses.

## Introduction

Different types of dying cells, including apoptotic cells, are removed from tissues to prevent immune reactions and maintain tissue homeostasis [1], [2], [3], [4]. Inability to recognize and remove dead cells can lead to diseases such as autoimmune disorders, cystic fibrosis, and asthma [5], [6]. The anti-inflammatory features of apoptotic cells resulting from surface exposure of anti-inflammatory molecules such as phosphatidylserine have been known for some time [7], [8]. These anti-inflammatory molecules are among the apoptotic cell-associated molecular patterns (ACAMPs) [9]. However, during the last couple of years it has become clear that apoptotic cells under certain conditions can also be immunogenic due to exposure/release of damage-associated molecular patterns (DAMPs) [10], [11]. A “danger theory” proposed by Matzinger states that the immune system can discriminate not only self from non-self but also dangerous signals (such as DAMPs) from innocuous ones [12]. DAMPs can be secreted, released and/or exposed on the outer leaflet of the plasma membrane and can provide several kinds of signals: ‘find-me’ (chemotactic), ‘eat-me’ (phagocytic), and ‘activation’ (immune stimulatory) factors [13].

DAMPs are recognized by membrane-bound or cytoplasmic pattern recognition receptors (PRRs), which include Toll-like receptors (TLRs), NOD-like receptors (NLRs), RIG-I-like receptors (RLRs) and purinergic receptors [14], [15]. Interestingly, cell death associated with autophagy can also provide immunogenic signals. It was recently shown that cross-priming of antigen-specific CD8+ T cells is facilitated when antigen donor cells undergo autophagy before dying by apoptosis [16]. Phagocytosis of MCF-7 cells dying by autophagy leads to inflammasome activation and IL-1β production in human monocyte derived macrophages [17], [18], but the autophagic dying cells can still inhibit the production of lipopolysaccharide (LPS)-induced pro-inflammatory cytokines (such as TNF-α, IL-6 and IL-8). Autophagy contributes to making apoptotic cancer cells immunogenic [19] and thereby capable of activating the inflammasome in dendritic cells [20]. However, the mechanism of inflammasome activation by dying autophagic cells is still not defined entirely.

IL-1β production is a tightly controlled process playing a pivotal role in inflammation and during recruitment of neutrophils into tissues [21]. A two-signal model has been proposed to explain the regulation of IL-1β production. First, pro-IL-1β is synthesized and accumulates in response to signaling through the TLRs, which usually activate the transcription factor known as nuclear factor kappa-light-chain-enhancer of activated B cells (NF-κB) and the activity of the IL-1β promoter [22]. A secondary stimulus (such as adenosine triphosphate (ATP) or DAMPs) induces the activation of cytoplasmic receptors. These nucleotide binding domain (NOD)-like receptors (e.g. NALP3) are normally auto-repressed, but their stimulation results in assembly of an inflammasome complex that recruits apoptosis-associated speck-like protein containing a caspase recruitment domain (ASC), which further recruits pro-caspase-1. Upon auto-cleavage of pro-caspase-1, its mature form cleaves pro-IL-1β and the cleaved IL-1β is secreted [23]. How NOD-like receptors sense the particular inducer and lead to secretion of IL-1β from macrophages has not been clarified in detail [24]. A common trigger of NALP3 inflammasome activation is a low intracellular potassium (K^+^) concentration, which occurs, for example, upon stimulation of macrophages by the ATP released during inflammation or tumor growth [19]; this ATP acts on purinergic receptor P_2_X_7_ [25], [26], [27], [28]. Opening of pannexin-1 channels, which has also been implicated in activation of the inflammasome pathway, results in cytosolic recognition of bacterial products in macrophages [29].

In the present work we have extended previous studies by gaining more insight into the mechanism of inflammasome activation by dying autophagic cells in various types of macrophages. We report that upon IL-3 withdrawal, pro-B lymphoma cells (Ba/F3) dying by apoptosis associated with autophagy become pro-inflammatory by inducing NALP3 inflammasome activation in the mouse macrophages engulfing them. We identified several elements of the upstream mechanisms of NALP3 activation, including secretion of ATP from the dying autophagic cells through pannexin-1 channels, activation of P_2_X_7_ receptors, and K^+^ efflux from the macrophages. We also show that pro-inflammatory dying autophagic cells recruit neutrophils *in vivo* and thereby induce an acute inflammatory response.

## Results

### During IL-3 Deprivation, Ba/F3 Cells Undergo Pronounced Autophagy Followed by Apoptosis

**Figure 1:**
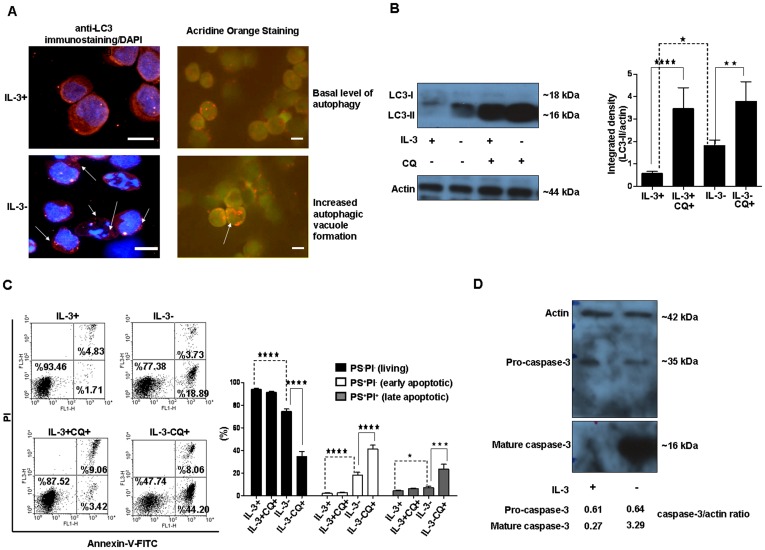
Autophagy is induced in apoptotic Ba/F3 cells by IL-3 depletion. Ba/F3 cells were kept without IL-3 for 6 h (dying AU). (**A**) Both living and dying autophagic Ba/F3 cells were stained with anti-LC3 antibody or acridine orange stain to demonstrate increased autophagosome formation. Arrows represent the increased autophagy with IL-3 depletion. Scale bars are 10 µm. (**B**) Proteins in western blots of samples from dying autophagic cells were detected with anti-LC3 antibody. Chloroquine (CQ) was used as lysosomal inhibitor. The right panel presents the quantification of the western blot. Data represent the mean ± SEM of 13, 19, 5 and 6 independent experiments for IL-3+, IL-3-, IL-3+CQ+ and IL-3-CQ+, respectively. (**C**) Cell death was quantified by flow cytometric analysis of dying autophagic cells by using Annexin-V-FITC/PI staining. Data represent the mean ± SEM of 7, 10, 3 and 9 independent experiments for IL-3+, IL-3−, IL-3+CQ+, IL-3−CQ+, respectively. PS: Phosphatidylserine, PI: Propidium iodide (**D**) Proteins obtained from dying autophagic cells were detected with caspase-3 antibody. Anti-actin poyclonal antibody was used to show that equal amounts of proteins were loaded in western blots. For simplicity, parts from the same western blots are shown separately in parts B and D. (*p<0.05, **p<0.01, ***p<0.001, ****p<0.0001).

Withdrawal of growth factors triggers both autophagy and apoptosis in Ba/F3 cells [30]. By using anti-LC3 immunostaining and acridine orange staining, we observed that 6 h of IL-3 depletion increased the numbers of autophagolysosomes in Ba/F3 cells (Figure 1A). Western blot analysis showed increased level of LC3-II (a molecular marker of autophagosome formation) relative to controls (Figure 1B). To determine whether IL-3 withdrawal causes upregulation of autophagosome formation (increased autophagic flux) or blockage of autophagic degradation, we treated the cells with the lysosomal inhibitor, chloroquine (CQ), which prevents fusion of autophagosomes with lysosomes [31], [32]. CQ treatment led to highly elevated LC3-II protein content in IL-3 depleted cells, demonstrating that withdrawal of the growth factor resulted in increased autophagic flux but not blockade of autophagy (Figure 1B). In the presence of IL-3, CQ treatment also led high LC3-II content indicating ongoing autophagic flux. It can not be excluded that since the cell suspensions are heterogeneous and the method is not sensitive enough there might be cells in which LC3-II degradation was blocked.

After 6 h of IL-3 withdrawal, ∼20% of the cells were positive for phosphatidylserine (PS)^+^ and negative for propidium iodide (PI)^-^, and ∼4% of them were PS^+^/PI^+^ (Figure 1C). This indicated initiation of apoptosis and is in accordance with findings showing caspase-3 processing (Figure 1D) after 6 h of IL-3 depletion [30]. When Ba/F3 cells were treated with CQ in the presence of IL-3, accumulation of autophagosomes did not lead to a significant increase in cell death, but the combined effect of IL-3 depletion and addition of the lysosomal inhibitor induced more cell death than IL-3 alone (Figure 1C).

### Apoptotic and necrotic cells die without increased signs of autophagy

**Figure 2:**
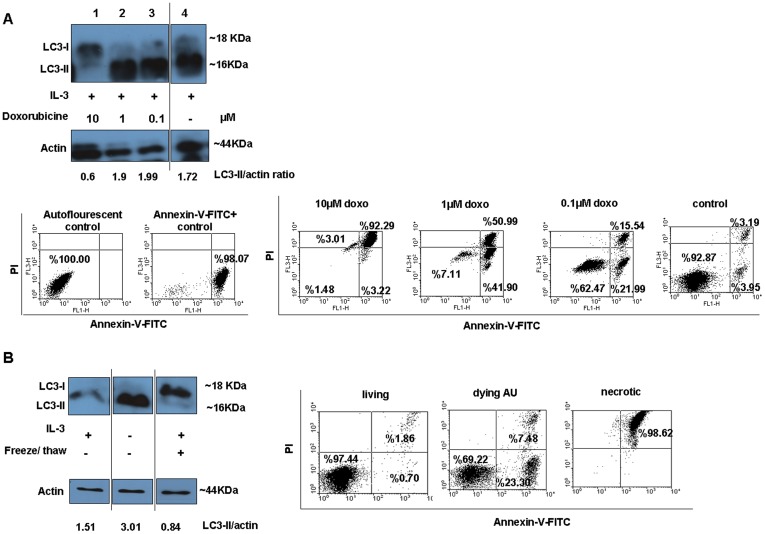
Apoptotic and necrotic Ba/F3 cells do not show autophagic activity. (**A**) Ba/F3 cells were treated with different doses of doxorubicin in the presence of IL-3 for 16 h. Immunoblotting with anti-LC3 antibody was done to detect LC3 protein in cells. Ba/F3 cells which were not treated with doxorubicin in IL-3 containing medium were used as control for the experiment. (**B**) Necrosis in Ba/F3 cells was induced by freeze-thaw. Anti-actin polyclonal antibody was used to show that equal amount of proteins were loaded. For simplicity, parts from the same western blots are shown separately. Cell death was checked by flow cytometric analysis of dying cells by using Annexin-V-FITC/PI staining. Relevant controls (autoflourescent and Annexin-V-FITC positive cells) are also included in the figure.

Ba/F3 cells were treated with various concentrations of doxorubicin in the presence of IL-3 for 16 h. According to our immunoblotting results, induction of apoptosis in Ba/F3 cells by 10 µM doxorubicin did increase their autophagic activity (Figure 2A). At lower doses of doxorubicin autophagy was increased but only a much lower percentage of cells died (Figure 2A). Necrotic Ba/F3 cells did not induce elevated autophagic activity neither (Figure 2B).

### Dying Autophagic Cells Induce Resident Macrophages to Release IL-1β, but Live, Apoptotic, Necrotic or Necrostatin-1-treated Cells do not

**Figure 3:**
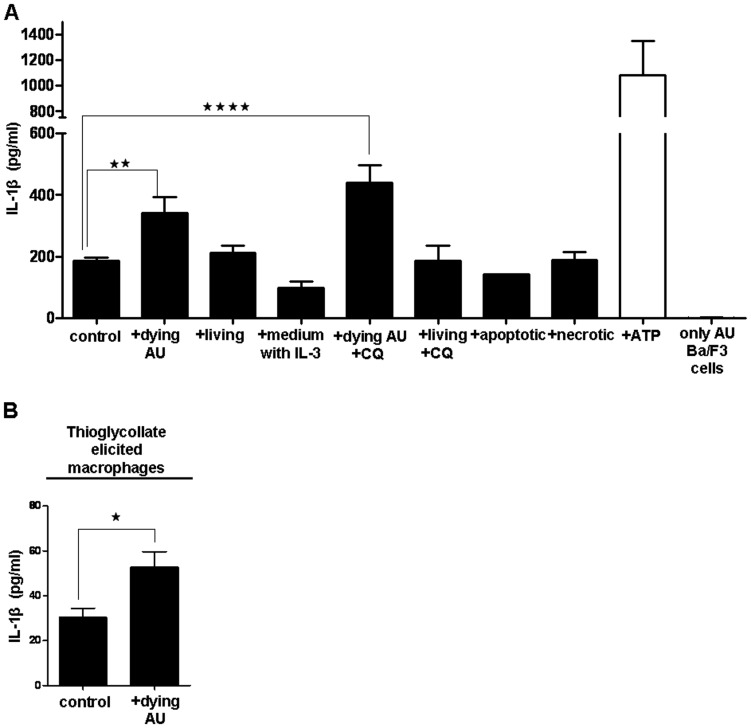
Resident peritoneal macrophages release IL-1β while engulfing dying autophagic Ba/F3 cells. (**A**) IL-3-depleted cells, live cells, apoptotic cells (treated with 10 µM doxorubicin), and necrotic Ba/F3 cells were co-incubated with primed resident macrophages. As a control condition, IL-1β was quantified from supernatant of only dying autophagic Ba/F3 cells in order to analyze if they secrete IL-1β by themselves. ATP, which is a stimulus for the inflammasome activation, was used as a positive control. (**B**) Primed thioglycollate-elicited macrophages were co-incubated with IL-3-depleted dying autophagic cells. Primed macrophages (control) but not co-incubated with any type of Ba/F3 cells. Unpaired non-bias two-tailed student t-test was used for statistical analysis in part B. Data represent the mean ± SEM of three and four independent experiments in parts A and B, respectively; all experiments were performed in triplicates. (*p<0.05, **p<0.01, ****p<0.0001).

Co-incubation assays were conducted to observe the response of macrophages to the presence of Ba/F3 cells. Non-primed resident peritoneal macrophages were unresponsive to dying autophagic Ba/F3 cells and released no IL-1β (data not shown) while LPS priming alone induced a basal levels of IL-1β secretion. When the macrophages were primed with ultrapure LPS to stimulate synthesis of pro-IL-1β before co-incubation with dying autophagic cells, secretion of mature IL-1β by macrophages was significantly increased (Figure 3A). IL-1β was released in even larger amounts when dying autophagic cells were treated with the lysosomal inhibitor (CQ) before their co-incubation with the macrophages (Figure 3A). Co-incubation of macrophages with live cells or with apoptotic (induced by 10 µM doxorubicin) or necrotic cells did not increase IL-1β secretion (Figure 3A). Thioglycollate-elicited macrophages (Figure 3B) and bone marrow derived macrophages (BMDMs) (data now shown) also released significantly more IL-1β than controls after their co-incubation with dying autophagic cells. Dying autophagic Ba/F3 cells by themselves did not produce IL-1β (Figure 3A), and culture medium used for Ba/F3 (with IL-3 and fetal bovine serum) did not induce IL-1β secretion in macrophages.

**Figure 4:**
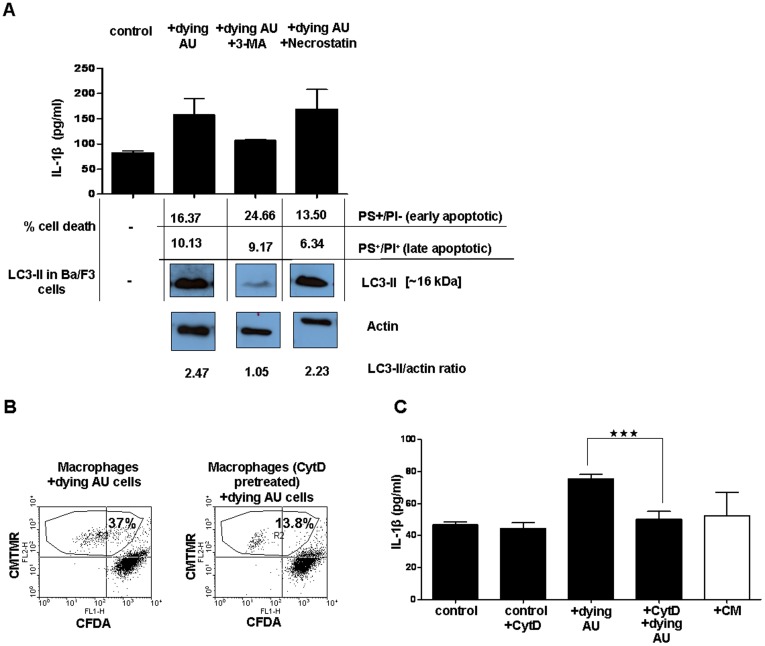
Uptake of dying autophagic cells and autophagic features of dying cells are required for macrophages to release IL-1β. (**A**) Primed resident macrophages were co-incubated with Ba/F3 cells that were IL-3-depleted, or IL-3-depleted and also pretreated with 3**-**methyladenine (3-MA) or necrostatin-1. Cell death was checked by flow cytometric analysis of dying autophagic cells by using Annexin-V-FITC/PI staining. For simplicity, parts of the same western blot are shown separately. (**B**, **C**) CMTMR-stained macrophages were treated with cytochalasin D (cytD) to inhibit phagocytosis, and cytD treated/non-treated macrophages were co-incubated with CFDA-stained dying autophagic Ba/F3 cells. R2 is the region which shows both the upper left quadrant (macrophages which do not engulf dying cells) and upper right quadrant (macrophages which engulf dying cells). The cells out of R2 region is also shown in the figure. The macrophages were also co-incubated with the conditioned medium (CM) from dying autophagic cells. In parts A and C, control cells are macrophages, which were primed but not co-incubated with any type of Ba/F3 cells. Data represent the mean ± SEM of two independent experiments; all experiments were performed in triplicates, (***p<0.001).

To determine whether autophagy in the phagocytosis target cells is required for IL-1β release from macrophages, we inhibited autophagy in Ba/F3 cells by the inhibitor of type III phosphoinositide 3-kinase (PI3K), 3-methyladenine (3-MA). Indeed, this inhibition decreased the pro-inflammatory response in the macrophages (Figure 4A). To exclude the involvement of the necroptotic cell death pathway, we treated the dying autophagic cells with the necroptosis inhibitor necrostatin-1 [2] and found that it could not prevent IL-1β release from resident macrophages exposed to dying autophagic cells (Figure 4A).

### Engulfment of Dying Autophagic Cells by Resident Macrophages is Required for IL-1β Release

Resident peritoneal macrophages engulfed 30% of dying autophagic cells and up to 43% of CQ-treated dying autophagic cells, in contrast to 12% of living cells and up to 27% of CQ-treated living cells during 2 h of co-incubation (data not shown). To investigate whether the uptake of dying autophagic cells by macrophages is important for IL-1β release, we pre-treated macrophages with cytochalasin D (CytD) to inhibit phagocytosis [33]. Indeed, CytD inhibited phagocytosis of dying autophagic cells (Figure 4B) and also significantly reduced IL-1β release. This indicates that the increased IL-1β secretion was dependent on the uptake of dying autophagic cells by macrophages (Figure 4C). Incubation of primed macrophages in conditioned medium (CM) obtained from cultures of Ba/F3 cells depleted of IL-3 for 6 h did not result in IL-1β release (Figure 4C).

### Dying Autophagic Cells Induce Caspase-1 Dependent NALP3 Inflammasome Activation in Thioglycollate-elicited Peritoneal Macrophages

**Figure 5:**
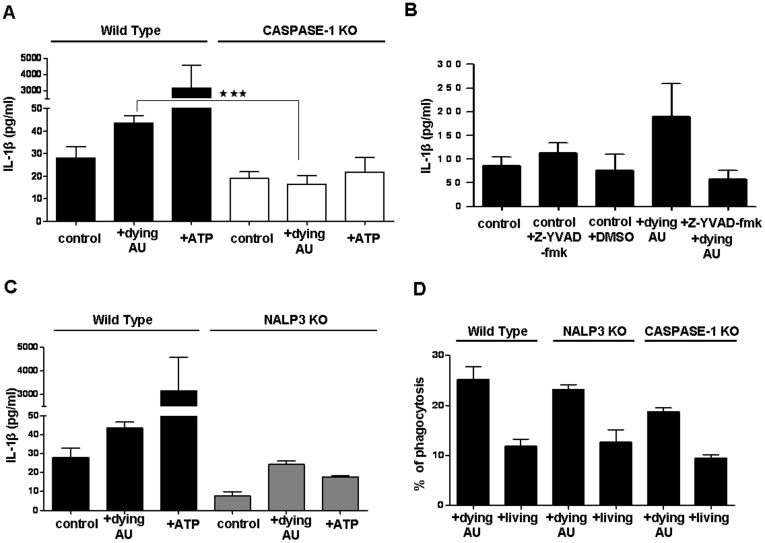
Uptake of dying autophagic cells leads to NALP3 and caspase-1 mediated IL-1β release in macrophages. Primed thioglycollate-elicited macrophages from wild type and from CASPASE-1 (**A**) or NALP3 (**C**) knockout mice were co-incubated with IL-3-depleted cells. ATP was used as a positive control. (**B**) Resident macrophages treated with Z-YVAD-fmk (specific caspase-1 inhibitor) were co-incubated with IL-3-depleted dying cells. (**D**) Wild type and NALP3 or CASPASE-1 deficient macrophages were co-incubated with IL-3 depleted dying autophagic (AU) and living Ba/F3 cells and phagocytosis was measured by flow cytometry. In parts A, B and C, control cells are primed macrophages but not incubated with any type of Ba/F3 cells. Data represent the mean ± SEM of two independent experiments for part A, three independent experiments for part B, one experiment for part C, and two independent experiments for part D; all experiments were performed in triplicates, (***p<0.001).

To check whether caspase-1 activation is responsible for the inflammatory response, we co-incubated caspase-1 knockout mouse macrophages with dying autophagic Ba/F3 cells. Caspase-1 deficient macrophages released significantly less IL-1β than wild type macrophages (Figure 5A). We confirmed this finding by using a specific caspase-1 inhibitor which also reduced IL-1β secretion (Z-YVAD-fmk, Figure 5B). Furthermore, macrophages isolated from NALP3 knockout mice had decreased response to dying autophagic cells, indicating that the NALP3 inflammasome can be the mediator of caspase-1 activation and IL-1β secretion (Figure 5C). There was no difference in the phagocytic capacity of wild type and NALP3 knockout macrophages (Figure 5D).

### Upstream Mechanisms of NALP3 Inflammasome Activation in Macrophages Engulfing Dying Autophagic Cells

**Figure 6:**
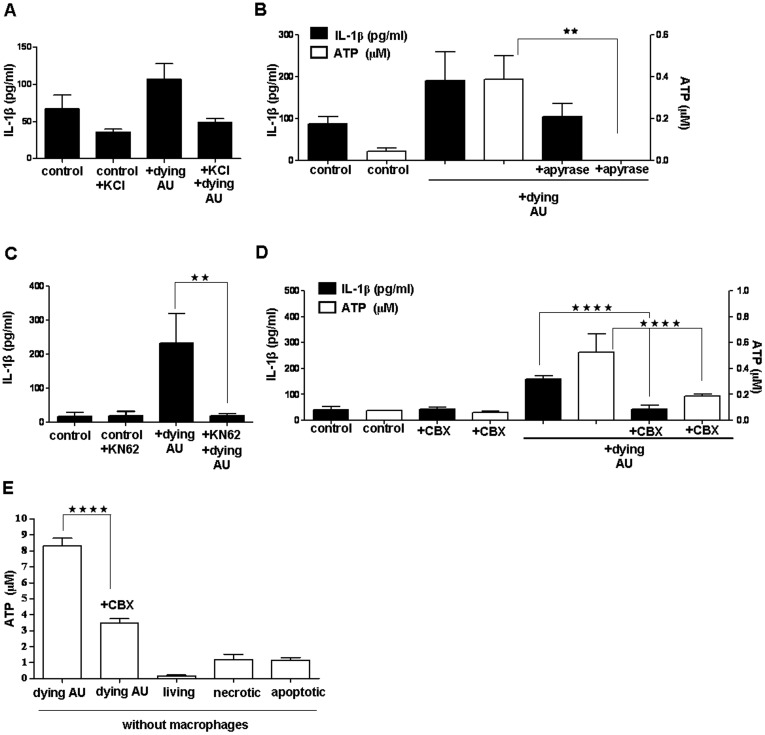
Analysis of upstream mechanisms of NALP3 inflammasome activation in peritoneal macrophages engulfing dying autophagic cells. (**A**) Primed resident macrophages were co-incubated with dying autophagic (AU) cells in the presence of KCl. Macrophages were treated with adenosine diphosphatase (apyrase) (**B**) and purinergic receptor inhibitor (KN-62) (**C**), or pannexin-1 channel inhibitor (CBX) (D) and co-incubated with IL-3-depleted dying autophagic cells. (**B**, **D**) ATP concentrations in culture media, (E) ATP concentrations in conditioned medium (CM) collected from Ba/F3 cells after 6 h of IL-3 depletion and CBX treated/non-treated dying AU cells (without macrophages). In parts A, B, C and D, control cells are primed macrophages but not co-incubated with any type of Ba/F3 cells. Data represent the mean ± SEM of pooled data from three experiments for A, two experiments for B, four experiments for C and D, and three experiments for E; experiments were performed in triplicates; (**p<0.01,**** p<0.0001).

We sought to identify the signaling pathway involved in NALP3 inflammasome activation triggered by engulfment of dying autophagic cells. We first investigated whether potassium efflux, a general inducer of NALP3 inflammasome activation [34], is required for induction of IL-1β release from mouse peritoneal macrophages by dying autophagic Ba/F3 cells. Blocking K^+^ efflux by including potassium in the medium led to decreased of IL-1β release from both resident peritoneal (Figure 6A) and thioglycollate-elicited peritoneal macrophages (data not shown).

The next question was whether ATP released during engulfment of dying autophagic cells contributes to inflammasome activation. A substantial amount of ATP (400–500 nM) was detected in the conditioned medium obtained after co-incubation of macrophages with dying autophagic cells in the absence of serum (Figure 6B). The hydrolysis of secreted ATP by apyrase during phagocytosis led to the decrease of IL-1β release (Figure 6B). These data indicate that released ATP contributed to the pro-inflammatory induction of macrophages. To further explore whether activation of the purinergic receptor P_2_X_7_ by released ATP is essential for inflammasome activation, we blocked the purinergic receptors by using 1-[N,O-bis(5-isoquinolinesulfonyl)-N-methyl-L-tyrosyl]-4-phenylpiperazine (KN-62) [35]. IL-1β release was inhibited by KN-62, indicating that NALP3 inflammasome activation was dependent on stimulation of the P_2_X_7_ receptor (Figure 6C).

We then analyzed the contribution of the pannexin-1 channel to inflammasome activation by using carbenoxolone (CBX), a specific pannexin-1 channel inhibitor [36], to block the channel’s activity during co-incubation of dying autophagic cells with macrophages. CBX treatment inhibited IL-1β release from resident macrophages (Figure 6D). Furthermore, CBX also blocked ATP secretion, showing that ATP was released through these channels (Figure 6D). To identify the source of the released ATP, we measured ATP released in the CM by dying autophagic cells cultured alone. We found that it was in the 8-10 µM range, and that its release could be inhibited by CBX (Figure 6E). Next, we checked whether living, necrotic and apoptotic Ba/F3 cells alone release ATP. We found that living cells did not release ATP, but necrotic and apoptotic Ba/F3 cells (10 µM doxorubicin) released ATP in the 1 µM range.

We also observed that when dying autophagic cells were removed from wells in which a co-incubation experiment has been conducted (after 2 h), macrophages did not release IL-1β any longer (data not shown), which indicates that ATP was released from dying cells but not from macrophages.

### Dying Autophagic Ba/F3 Cells Recruit Neutrophils into the Peritoneal Cavity as a Sign of Acute Inflammatory Response

**Figure 7:**
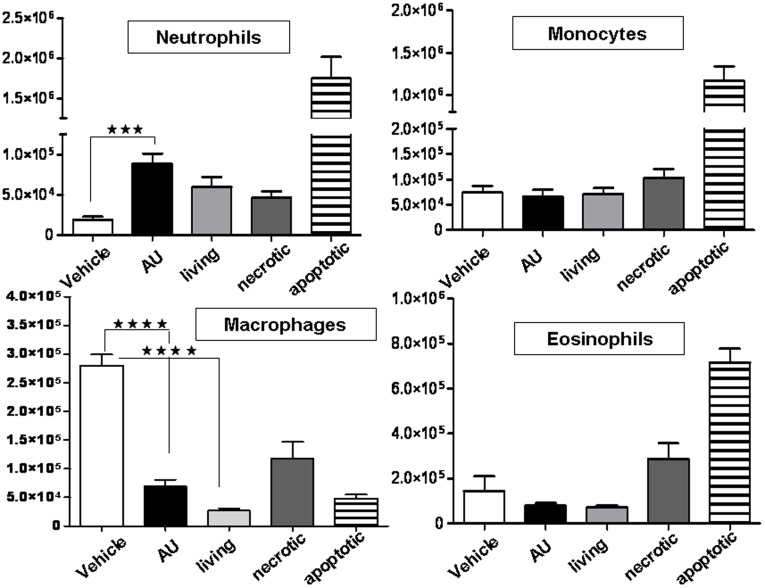
Intraperitoneal injection of IL-3-depleted dying autophagic Ba/F3 cells induces a sterile inflammatory response (neutrophil influx). Dying autophagic, apoptotic or necrotic cells and living cells were intraperitoneally injected into wild type BALB/c mice. Equal volumes of D-PBS were injected in mice as negative controls. Peritoneal exudate cells (PECs) were collected 16 h later and monocytes, macrophages, eosinophils and neutrophils were stained with anti-mouse antibodies F4/80-APC with CD11b-APC-Cy7 and Ly-6G-APC with CD11b-APC-Cy7 and analyzed on BD LSR-II. Graphs represent the number of macrophages (F4/80^high^ CD11b^high^), monocytes (F4/80^medium^ CD11b^high^), eosinophils (F4/80^medium^ CD11b^medium^) and neutrophils (CD11b^+^ Ly6G^high^) in PECs after injection of AU, necrotic, living or apoptotic Ba/F3 cells. (***p<0.001, ****p<0.0001).

In our attempt to support our *in vitro* results showing that dying autophagic Ba/F3 cells are pro-inflammatory, we injected dying autophagic cells into the peritoneum of mice. We observed influx of neutrophils into the peritoneal cavity, indicating that the dying autophagic cells induced an acute inflammatory response *in vivo* as well (Figure 7). Living, necrotic and apoptotic Ba/F3 cells (10 µM doxorubicin) were also injected i.p. Apoptotic cells recruited neutrophils, monocytes and eosinophils, but they led to the decrease of macrophages resident in the peritoneum. Living, autophagic and necrotic cells also diminished the number of macrophages. Necrotic and living cells could not induce neutrophil influx. Only necrotic and apoptotic cells recruited eosinophils into the peritoneal cavity.

### Dying Autophagic Cells Inhibit Crude LPS-induced IL-6 Release

**Figure 8:**
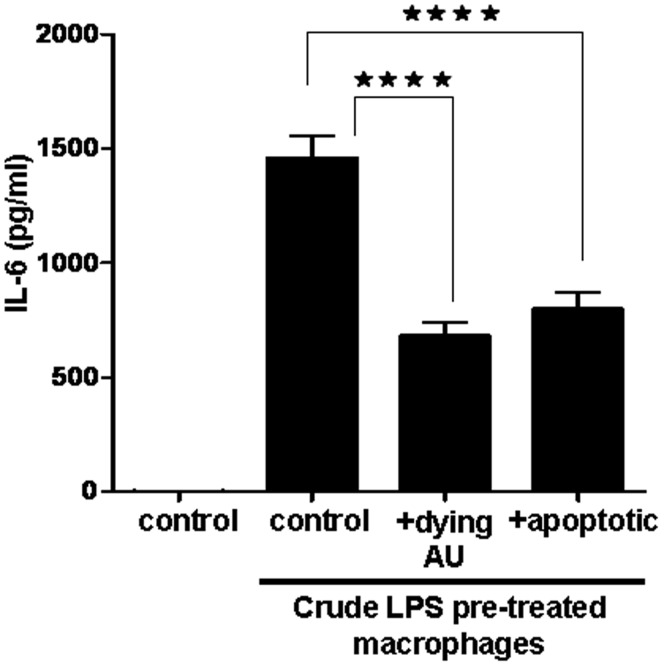
Crude LPS-induced IL-6 release from macrophages is inhibited by dying autophagic cells Thioglycollate-elicited peritoneal macrophages pre-treated with crude LPS were co-incubated with IL-3-depleted dying autophagic (AU) cells or with doxorubicin treated apoptotic cells. Data represent the mean ± SEM of pooled data from one experiment performed in five replicates; (****p<0.0001).

Although the dying autophagic Ba/F3 cells could induce a pro-inflammatory response in macrophages and provoked them to secrete IL-1β, they could also inhibit the LPS-induced pro-inflammatory response as measured by IL-6 release (Figure 8).

## Discussion

We have shown that mouse macrophages display a pro-inflammatory response to Ba/F3 lymphoma cells dying by apoptosis associated with autophagy in response to IL-3 withdrawal, but not when they are exposed to living, apoptotic, necrotic or necrostatin-1-treated cells. The NALP3 inflammasome and caspase-1 were activated in the macrophages while they were taking up dying autophagic cells, leading to IL-1β release from the macrophages. Activation of NALP3 was dependent on the uptake of dying cells, ATP release through the pannexin-1 channel (P_2_X_7_ purinergic receptor), and K^+^ efflux. Injection of dying autophagic cells intraperitoneally induced an acute inflammation by recruiting neutrophils.

Increased autophagy after IL-3 depletion was demonstrated in dying Ba/F3 cells by acridine orange staining and punctuate pattern which represents autophagosomes detected with anti-LC3 antibody, as well as by the increased level of LC3-II. These data are in line with previously published results obtained with Ba/F3 cells, which showed that IL-3 dependent murine Ba/F3 pro-B cells respond to growth factor withdrawal by inducing autophagy as a survival mechanism [30]. Moreover, it has been shown that increased autophagic activity sensitizes Ba/F3 cells for apoptosis through caspase-dependent generation of beclin-1 cleavage fragments and degradation of type III PI3K [30]. IL-3 withdrawal leads Ba/F3 cells to stay in G0/G1 phase [37] and the most apoptosis-resistant Ba/F3 cells can use autophagy-derived nutrients under growth factor depletion. However, autophagic activity can be a sensitizer for these cells and they become more prone to die by apoptosis [38]. We wanted to clarify that IL-3 depletion leads to upregulation of autophagy instead of blockage of autophagy (degradation block). Since LC3-II is also degraded by autophagy increased amount of LC3-II protein by itself at a certain time can not represent properly the dynamics of autophagy and it is recommended to observe LC3 levels following lysosomal inhibition [31], [32]. We have treated our IL-3 depleted/non-depleted Ba/F3 cells with lysosomal inhibitor (CQ) and found that blocking the fusion of autophagosomes with lysosomes by CQ treatment led to accumulation of LC3-II, which further proved that the increased autophagy after IL-3 withdrawal was due to up-regulation of autophagosome formation (autophagic flux) and not to lysosomal blockage. According to our results, there is a basal autophagy rate in Ba/F3 cells. When we added CQ to living cells (IL-3+), LC3-II accumulated due to blocking of the basal autophagic activity, but there was no increase in cell death (Figure 1B, 1C). On the other hand, IL-3 depletion led to significant elevation of autophagic activity above the basal level and to cell death. We have also shown that blockage of the lysosomal pathway by CQ treatment increases the percentage of cell death when the cells are depleted of IL-3.

We show that uptake of dying autophagic Ba/F3 cells by different types of murine macrophages activates the NALP3 inflammasome pathway. This is in line with our previous findings from experiments in which we used human MCF-7 cells dying by autophagy as phagocytosis targets of monocyte derived macrophages [17], [39]. Despite the similarities between our previous and current studies, important differences and new aspects have been revealed. Mouse macrophages have to be primed in order to induce pro-IL-1β production, whereas in human macrophages the dying autophagic MCF-7 cells could both up-regulate the expression of the inflammasome system and activate it in the absence of a priming signal. In our current study, we have shown that different types of primed mouse primary macrophages (resident, bone marrow-derived and elicited) can respond by IL-1β release to dying autophagic Ba/F3 cells. We also showed in an *in vivo* mouse model the pro-inflammatory characteristics of dying autophagic Ba/F3 cells. Furthermore, unlike the autophagic MCF-7 cells that die due to induction of autophagy after starvation and tamoxifen treatment [40], IL-3 depleted Ba/F3 cells are not killed but only sensitized for apoptosis by autophagy. Finally, we have shown in both our previous and current studies that released ATP leading to purinergic receptor activation and subsequent K^+^ efflux induces NALP3 inflammasome activation in both human monocyte-derived macrophages and primed peritoneal mouse macrophages. However, ATP was released from human monocyte-derived macrophages during phagocytosis of autophagic dying MCF-7 cells [17], whereas in our present study we demonstrated that ATP was released from dying autophagic cells, not from macrophages.

Apoptotic cell death is induced in Ba/F3 cells by the cleavage of the autophagy protein, beclin-1, which leads to release of cytochrome c from mitochondria during IL-3 depletion [30]. Indeed, it has been shown [30] that at the onset of apoptosis, about 6 h after IL-3 withdrawal, it is not the high autophagy rate that kills the cells. When MCF-7 or Ba/F3 cells die by apoptosis, necrosis or necroptosis, their engulfment does not lead to inflammasome activation. This indicates that only cells dying with autophagic features due to growth factor depletion lead to inflammasome activation in macrophages. Though 3-MA is a non-specific chemical inhibitor for autophagy we can see the decrease in IL-1β release from macrophages which is co-incubated with autophagy inhibited dying cells. Of note, doxorubicin-treated apoptotic Ba/F3 cells could not induce inflammasome activation probably because they died by apoptosis without up-regulating autophagy. On the other hand, it has been shown that doxorubicin induces immunogenic cell death in cancer cells through the calreticulin exposure pathway as well as inflammasome activation in the phagocytic cells [41]. In our previous work, knocking down calreticulin in autophagic dying cells did not prevent inflammasome activation in macrophages taking up these cells [17]. Another recent study demonstrated that doxorubicin-induced immunogenic apoptotic cells can be recognized and responded to by the TLR-2/TLR-9-Myeloid differentiation primary response gene (88) (Myd88) signaling pathway; this finding provides an alternative explanation for their pro-inflammatory effect [11].

It was recently shown that accumulation of autophagic vacuoles in glioblastoma cell lines following treatment with a lysosome inhibitor [42] results in a sustained imbalance: the rate of autophagic vacuole formation exceeds the rate of autophagic vacuole degradation and promotes development of autophagic stress predisposing to neuronal and glial cell death [43]. In our current study, we checked whether dying autophagic Ba/F3 cells with autophagic stress due to autophagosome accumulation can also lead to an inflammatory response in macrophages. Autophagosome accumulation by itself, induced by CQ treatment in the presence of IL-3, did not induce cell death and was not sufficient to cause caspase-1 activation when these cells were engulfed. Even increased autophagy in dying cells, which can occur, for example, during surface detachment or anoikis in MCF-7 cells [40], does not induce inflammasome activation [17]. Therefore, it appears that cell death of target cells has to be initiated by autophagy (or at least autophagy has to sensitize cells for apoptosis) to create the molecular pattern needed for inflammasome activation following phagocytosis of these cells. This conclusion is supported by the finding that a combination of IL-3 depletion and lysosomal inhibitor treatment promotes higher rates of cell death and leads to more efficient engulfment of dying cells and stronger induction of the inflammasome activating pathway, together with the release of more IL-1β from the engulfing macrophages.

We have shown that IL-1β release from macrophages co-incubated with dying autophagic cells is caspase-1 dependent by using specific caspase-1 inhibition and caspase-1 deficient macrophages. We then checked whether the NALP-3 inflammasome plays a role in this pro-inflammatory response. When we co-incubated NALP-3 deficient macrophages with dying autophagic cells, the released IL-1β was less than from control macrophages but a lower level of inflammasome activation was still observed. Even the known NALP-3 inducer ATP mediated IL1-β release [23] from NALP-3 deficient macrophages was not completely prevented either. Ultra-pure LPS priming of macrophages also led to weaker inflammasome activation in the knockout macrophages. We do not claim that only NALP-3 inflammasome can be activated in macrophages while engulfing dying autophagic cells. Inflammasome complexes other than NALP-3 might also be activated by engulfed autophagic dying cells, especially in mice developing in and compensating for the absence of NALP-3. It was recently shown that there is cooperation between NLRP3 and NLRC4 inflammasomes *in vivo* during *S. typhimurium* infection, and that deficiency of either NLRP3 or NLRC4 does not change the bacterial infection in the mice [44], [45]. On the other hand, caspase-1 activity in NLRP3-deficient obese mice decreases but is not abolished in the presence of inflammasome inducers [44], [46], suggesting that inflammasome “priming” [47] plays a role at least in the pathophysiology of obesity. Additionally, it is also known that components of the NLRP1 and NLRC4 inflammasomes are constitutively expressed in cells, whereas NLRP3 transcription is triggered by bacterial components through the TLR4 pathway, a process that is also called “priming” [47].

Classical apoptotic cells are strong inhibitors of the TLR-mediated, NF-κB dependent inflammatory response of macrophages [7]. Accordingly, as we demonstrate here, dying autophagic Ba/F3 cells can also inhibit LPS-triggered IL-6 secretion. This well known anti-inflammatory effect of apoptotic cells is mediated by cell surface interactions and does not require phagocytosis of dying cells [7]. We found that dying autophagic Ba/F3 cells had to be internalized by macrophages to induce inflammasome activation and IL-1β release, which do not depend on the NF-κB pathway. The engulfed dying autophagic cells had to initiate an intracellular signaling pathway to induce inflammasome activation and exert pro-inflammatory effects. It is not clear how the engulfment process and/or the specific components of the dying autophagic cells sensitize macrophages for the ATP-dependent NALP3 activation.

Several mechanisms that are not mutually exclusive have been proposed to explain how NALP3 inflammasomes are activated. Direct interaction between NALP3 and its activators has been shown only in a few cases. For instance, bacterial muramyl dipeptide (MDP) and bacterial cell wall peptidoglycans interact directly with the leucine-rich repeat (LRR) part of NALP1 and NALP3, respectively [48]. However, NALP3 inflammasome activation pathways have not been defined for most pathogen-associated molecular patterns (PAMPs) and DAMPs, and it is unlikely that the different activators are specifically sensed by the inflammasome. One of the general mechanisms of inflammasome activation involves extracellular ATP, which generates an activation signal via the purinergic P_2_X_7_ receptors, followed by rapid K^+^ efflux leading to low intracellular K^+^ levels. AT-mediated activation of P_2_X_7_ can trigger K^+^ efflux from the cytosol [49]. Physiologic concentrations of intracellular K^+^ can prevent inflammasome assembly, and monosodium urate crystals can lead to inflammasome activation by causing K^+^ efflux from macrophages. Based on these observations, it has been proposed that a lowered K^+^ concentration in the cell is a common trigger of inflammasome activation [26]. Here, we demonstrate that this also takes place when dying autophagic cells are taken up by macrophages. In our experiments, inflammasome activation by dying autophagic cells could be suppressed by incubating macrophages with dying cells in a medium containing a high concentration of K^+^, which prevents K^+^ efflux. Inhibition of K^+^ efflux also decreased the basal levels of IL-1β released from ultra-pure LPS pre-treated macrophages. Thus, during recognition and engulfment of dying autophagic cells by macrophages, ATP released in the extracellular space might initiate the above-described sequence of events. Indeed, we found that a substantial amount of ATP was released during co-incubation of dying cells with macrophages. Hydrolyzing ATP by apyrase or blocking the P_2_X_7_ receptor by a specific antagonist during phagocytosis of dead cells significantly reduced IL-1β secretion. ATP is a known NALP3 agonist, and different PAMPs and DAMPs have been shown to lead to ATP release from monocytes followed by autocrine stimulation of purinergic receptors such as P_2_X_7_ [35]. ATP released from dying tumor cells acts on P_2_X_7_ purinergic receptors of dendritic cells, which can lead to inflammasome activation and further IL-1β secretion [20]. It was recently shown that certain types of necrotic cells can also release ATP and activate the NALP3 inflammasome in engulfing macrophages [50]. In our experiments, necrostatin-1 treatment of dying Ba/F3 cells did not prevent inflammasome activation during engulfment. This shows that necroptosis was not involved in this pro-inflammatory cell death process.

Blocking pannexin-1 channels during co-incubation of macrophages with dying autophagic cells led to inhibition of ATP release and inflammasome activation, which indicates that this channel was involved in ATP secretion. When we used Ba/F3 cells dying due to the absence of IL-3 but in the presence of serum, we could not detect ATP in the conditioned medium. However, ATP was secreted from the dying cells while they were incubated in the absence of macrophages and serum and this could be inhibited by the pannexin-1 channel blocker. This is in line with other studies in different model systems. The pannexin-1 channel was identified as a plasma membrane channel mediating the regulated release of ATP and UTP (both are “find me” signals for phagocytes) from apoptotic cells; activation of pannexin-1 is caspase 3 dependent [51]. It was recently shown that the autophagy-dependent release of ATP from dying tumor cells treated with anti-cancer agents made these cells immunogenic [19]. It was reported that LPS-treated peritoneal macrophages could release IL-1β in response to ATP in a pannexin-1 channel dependent way [52]. Using short hairpin (sh)RNA to silence pannexin-1 channels in neurons and astrocytes, it was also demonstrated that pannexin-1 channels are needed for inflammasome activation [53]. Inhibition of the pannexin-1 channel in J774 macrophages showed that the pannexin-1 pathway is essential for caspase-1 activation and mature IL-1β release [29], [36]. On the other hand, in macrophages of pannexin-1 deficient mice, most of the known inflammasome activators can elicit caspase-1 activation and IL-1β maturation and secretion [54], indicating that pannexin-1 is dispensable for the assembly of caspase-1 mediated complexes. However, these mice are deficient in ATP release from cells, including apoptotic cells, and this deficiency is in line with several studies on various cell types showing that this channel might be responsible for ATP release [55].

We observed that 8–9 µM ATP is released from dying autophagic Ba/F3 cells when they are incubated alone, but the concentration of released ATP is only 0.4–0.6 µM during the co-incubation of ultra-pure LPS primed macrophages with dying autophagic Ba/F3 cells. It is possible that ecto-ATPases were present in the medium during the co-incubation, which would diminish the amount of ATP released from dying autophagic cells. When we checked whether living, necrotic and apoptotic Ba/F3 cells release ATP, we observed that living cells did not release ATP whereas necrotic and apoptotic cells secreted about 1 µM ATP when they were not co-incubated with macrophages. Since necrotic and apoptotic cells could not up-regulate IL-1β release from macrophages, we assume that this small amount of ATP is also neutralized by ecto-ATPases, preventing activation of the purinergic receptors.

Treatment of cancer cells with anticancer chemicals (such as oxiplatin and mitoxantrone) causes cancer cells to die in an immunogenic manner [19]. It has been shown that dying cells are autophagic and induce an immunogenic response *in vivo* by recruiting dendritic cells and T cells into the tumor by releasing ATP into the extracellular fluid [19]. The authors also indicated that the immunogenicity of dying cells depends on autophagy mediated release of ATP [19]. According to our results, both ATP released from dying autophagic Ba/F3 cells and phagocytosis of dying autophagic cells play a role in inflammasome activation in macrophages. Furthermore, such an immunogenic response can also be elicited when autophagy and cell death is induced by cytokine depletion. When we treated Ba/F3 cells with doxorubicin, which is also a known immunogenic anticancer drug, less ATP was released than from autophagic dying cells, and this smaller amount was not sufficient to induce inflammasome activation *in vitro.* As we discussed above, this is likely due to ecto-ATPases and raises the possibility that regulation of the intensity of autophagy and thereby ATP release in dying tumor cells might be important for achieving an effective immunogenic response in the host, like the effect of increasing ATP levels in the tumor environment by inhibiting ecto-ATPases [19].

Our *in vivo* results show that neutrophil influx (a sign of an acute inflammatory response) was triggered by injection of dying autophagic Ba/F3 cells in the peritoneal cavity of mice. Both viable and necrotic Ba/F3 cells could recruit neutrophils into the peritoneum, though to a lesser extent than autophagic dying ones. It is very likely that injected “viable” cells start to die with increased autophagy due to the lack of IL-3 cytokine in the peritoneal cavity. Necrotic cells were frozen-thawed only once, and residual intact cells cellular parts could have been destroyed in the peritoneal cavity. Release of the contents of these newly destroyed cells could recruit neutrophils and eosinophils to the peritoneal cavity. Of note, doxorubicin-killed apoptotic Ba/F3 cells were the most potent inducers of neutrophil attraction in the peritoneum. This is understandable because doxorubicin-treated apoptotic cells have been shown to be the most potent inducers of acute inflammation *in vivo* in several models. Injection of doxorubicin into the peritoneal cavity of mice triggers a rapid neutrophil influx that is associated with the apoptosis of monocytes/macrophages [11]. Therefore, when doxorubicin-killed cells are injected into the peritoneum, doxorubicin leaking from the apoptotic cells might induce the death of peritoneal cells. This effect can lead to the recruitment of immune cells to the peritoneum (e.g. neutrophils) as shown by Krysko et al. [11]. Another study has shown that an immunogenic form of apoptosis was induced by mitoxantrone, another prototype of anthracyclines [10], [56]. Stimulation of cancer cells with mitoxantrone results in recruitment of dendritic cells and T lymphocytes to the site of the tumor bed [19]. It has also been shown that this property of dying cancer cells depends on their autophagic features, but this is not relevant for our data because we have shown that apoptotic Ba/F3 cells treated with 10 µM doxorubicin do not exhibit autophagic features. Therefore, it is likely that cells (autophagic dying cells or apoptotic or necrotic cells) that we injected contained danger signals independent of their dying status and that these signals could induce an acute inflammatory response.

In summary, our findings point to mechanisms that lead to NALP3 inflammasome activation in macrophages engulfing pro-inflammatory dying autophagic cells. These findings contribute to our understanding of how cancer cells can become immunogenic. DAMPs exposed on dying autophagic cells and triggering inflammasome activation inside macrophages have not been fully clarified, and our future studies will focus on identifying the molecular pattern associated with autophagic cells. That knowledge might make it possible to therapeutically target autophagy in cancer and other diseases.

## Materials and Methods

### Isolation of Macrophages

C57BL/6 mice, 6–9 weeks old, were used in all experiments unless otherwise specified. Animals were maintained in the pathogen-free animal facility of University of Debrecen (Debrecen, Hungary) and at the Department for Molecular Biomedical Research of Ghent University-VIB under the guidelines and ethically approved protocols of Ghent University and VIB. Peritoneal macrophages were obtained by peritoneal lavage from mice that were either injected with 2 ml of 4% thioglycollate or not injected. Thioglycollate-elicited peritoneal macrophages were collected from the peritoneal cavity of mice three days after injection. These macrophages were obtained from wild type and from mice deficient in *NALP3* (C57BL/6 background) or *Caspase-1* (6x back crossed to C57Bl/6). For experiments with knockout mice, wild type mice of appropriate background were used as controls, and they were bred under the same animal house conditions as the others. Macrophages from some mice were pooled and cells were collected by centrifugation and plated in 96-well plates (Corning, Lowell, MA) at 3x10^5^ cells per well in RPMI-1640 medium (Sigma, Steinheim, Germany) supplemented in 10% heat-inactivated fetal calf serum (FCS), 300 mg/L L-glutamine (Sigma), 100 U/ml penicilline/0.1 mg/ml streptomycin (Sigma), and 1 mM sodium pyruvate (Sigma). They were incubated at 37°C in an air atmosphere containing 5% CO_2_. Macrophages were used for co-incubation experiments on the third day after collection from the peritoneal cavity. Each day, unattached cells were removed by refreshing the medium.

Bone marrow derived macrophages (BMDMs) were differentiated from femoral bone marrow cells with 10% L929 conditioned medium in RPMI medium (Sigma). Every other day, the medium was replaced with the fresh medium. On the sixth day, cells were collected with enzyme free cell dissociation buffer (Gibco, Budapest, Hungary) and plated in a 96-well plate. They were used for the co-incubation assay on the third day.

### Induction of Different Types of Cell Death in Ba/F3 Cells

Ba/F3 cells are an IL-3-dependent pro-B cell line derived from mouse bone marrow. It is an established cell line that is used in Peter Vandenabeele’s Molecular Signalling and Cell Death Unit, Department for Molecular Biomedical Research, VIB, Ghent, Belgium. The same cell line was used also in the experiments reported in [30]. Ba/F3 cells were grown in RPMI-1640 supplemented with 10% heat-inactivated FCS (Sigma), 10% conditioned medium from WEHI-3B cells (a source of murine IL-3), 300 mg/L L-glutamine (Sigma), 100 U/ml penicillin and 0.1 mg/ml streptomycin (Sigma), 400 µM sodium pyruvate (Sigma), and 50 µM β-mercapto-ethanol (Sigma). They were incubated at 37°C in a humidified air atmosphere containing 5% CO_2_. All cell lines were *Mycoplasma* free. For cell death induction, Ba/F3 cells were resuspended in fresh medium at 2×10^4^cells/ml, and on the next day IL-3 was depleted for 6 h to induce autophagic death. IL-3-depleted and non-depleted cells were treated with chloroquine diphosphate (CQ-25 µM) (Fluka, Buchs SG, Switzerland**)**. Apoptotic cell death was induced by adding 10 µM doxorubicin (Sigma) for 16 h as described by Wirawan E. et al. [30]. Different concentrations of doxorubicin were used to optimize the apoptotic cell death conditions for Ba/F3 cells so that autophagic activity was not induced. Necrotic cells were prepared by freezing and thawing. After cells were thawed, they were washed with PBS and used in the experiments. To block necroptotic components, necrostatin-1 (Sigma) was used at 30 µM.

### Cell Death Assay and Autophagy Detection by Fluorescent Microscopy

Cell death was assessed by the Annexin-V fluorescein isothio-cyanate Apoptosis Detection Kit (MBL, Budapest, Hungary) according to manufacturer’s instructions. Propodium iodide (PI) staining was used to determine plasma membrane permeability. Percent of cells positive for Annexin-V or PI was determined on a FACSCalibur flow cytometer (BD FACSCalibur™ flow cytometer, Franklin Lakes, USA).

Autophagosome formation was visualized by fluorescent microscopy (Axiovert-150 Zeiss, Budapest, Hungary) by staining dying autophagic cells with anti-LC3 antibody and acridine orange (Sigma) (1 µM, 20 min). For staining with LC-3 antibody and visualization of autophagosomes living and IL-3 depleted Ba/F3 cells, cytospins of Ba/F3 cells were fixed with 4% paraformaldehyde in PBS for 15 min. Blocking was done with 5% BSA (bovine serum albumin) in PBS + %0.1 Triton-X for 1 h. They were then incubated with anti-LC3 polyclonal antibody (5 µg/ml, Novus Biologicals, Cambridge, England) at room temperature for 2 h. Secondary antibody was Cy3-labeled goat anti-rabbit (Sigma) and was used for 1 h. Nuclei were labeled with DAPI (Sigma) and viewed with a fluorescent microscope (Axiovert-150 Zeiss, Budapest, Hungary). Washings were done for 3×5 min with PBS containing %0.1 Triton-X.

### Immunoblot Analysis

The anti-LC3 polyclonal antibody mentioned above was also used in immunoblot analysis. Equal amounts of proteins (17.5 µg) obtained from cell lysates were separated on a NuPAGE 15% Bis-Tris polyacrylamide gel (Invitrogen, Merelbeke, Belgium) and transferred to an Immobilon-P membrane (Millipore, Budapest, Hungary; pore size 0.45 µm). Membranes were blocked in Tris buffered saline containing 0.05% Tween-20 (TBS-T) and 5% non-fat dry milk (BioRad, Budapest, Hungary) for 1 h. After blocking, membranes were probed overnight at 4°C with anti-LC3 polyclonal antibody (2 µg/ml; NovusBiologicals) and anti-actin polyclonal antibody (0.8 µg/ml) (Sigma) followed by incubation for 1 h with a rabbit anti-rat peroxidase-conjugated secondary antibody (Sigma) for 1 h at room temperature. Pro- and mature caspase 3 (1 µg/ml) were also checked by using caspase 3 antibody (BD Pharmingen, Budapest, Hungary). Peroxidase activity was detected with SuperSignal West Femto Maximum Sensitivity Chemiluminescent Substrate (Pierce, Rockford, IL) using a Lumi-Imager (Roche Diagnostics, Mannheim, Germany). Fermentas pre-stained protein ladder was used as protein marker in each blot. Blots in Figure 1B, 1D and 2A, we have not de-stripped the membrane before detecting the actin protein and we have developed the membrane with anti-actin antibody after the washing steps upon developing it for anti-LC3. In Figure 2B and 4A, membranes were de-stripped (15-20 min), washed 3-4 X for 10 min with TBS-T and then re-blocked with 5% non-fat dry milk in TBS-T solution for 1h at room temperature. Then membranes were probed overnight with anti-actin first antibody, then the anti-rabbit secondary antibody for 1 h at room temperature. Detection of the peroxidase activity was done same as explained above. Stripping solution contains 2% SDS, 100 mM beta-mercaptoethanol and 50 mM TRIS, pH 6.8. The ratio of the integrated density of LC3-II to actin was quantified by using Image J (NIH Bethesda).

### *In vitro* Phagocytosis Assay

Thioglycollate-elicited peritoneal macrophages, resident macrophages and BMDMs were collected and plated as described above. They were primed with ultra-pure *E. coli* LPS (Invivogen, Toulouse, France) (0.05 ng/ml for resident macrophages, 500 ng/ml for thioglycollate-elicited macrophages, 100 ng/ml for BMDMs) 4 h before starting the phagocytosis assay. The ratio of phagocytes (3×10^5^cells/well) to cells to be engulfed (1.5×10^6^cells/well) was set at 1∶5. Macrophages were labeled with 5-(and-6)-(((4- chloromethyl)benzoyl)amino)tetramethylrhodamine (CMTMR) (Invitrogen) (7.5 µM, 4 h) and dying cells were stained with 5-(and-6)-carboxyfluorescein diacetate, succinimidyl ester (CFDA) (Invitrogen) (17.5 µM, 6 h). The phagocytosis assay was started when the cells to be engulfed were added to the phagocytes and they were kept together for 2 h. After the phagocytosis assay and following removal of non-engulfed dying cells, macrophages were treated with trypsin at 37°C for 15 min to remove attached cells so that only the macrophages engulfing dying cells are quantified.

The net phagocytosis rate was always checked by FACS analysis. Dying autophagic, apoptotic or necrotic cells and living cells were fed to macrophages and co-incubated for 2 h. Addition of 5 mM ATP (Sigma) was used as a positive control for IL-1β. Living Ba/F3 cells were fed to macrophages in their complete medium. When effect of inhibition of autophagy with 10 mM 3-methyladenine (Sigma) and effect of inhibition of necroptosis with 30 µM necrostatin-1 (Sigma) were investigated, they were added to the cultures during the 6 h of IL-3 depletion. Inhibition of phagocytosis was carried out by pre-treating the macrophages with 0.1 µM cytochalasin D (CytD) (Sigma) for 45 min at 37°C and maintaining this treatment throughout the phagocytosis assay. Studies on the role of P_2_X_7_R activation was carried with the ATP-hydrolyzing apyrase (Sigma) (2.5 units/ml) and the P_2_X_7_ receptor antagonist KN-62 (Sigma) (1 µM) (apyrase and KN-62 treatments were done 45 min before and throughout the phagocytosis assay). The role of the pannexin-1 channel in NALP3 activation was checked by using carbenoxolone disodium salt (CBX) (Sigma) (5 µM) 45 min before and throughout the phagocytosis assay. Dying autophagic cells were also treated with CBX (5 µM). Studies on the role of potassium efflux were carried out by using medium containing 130 mM KCl (Sigma) during co-incubation. Studies on how the specific caspase-1 inhibitor Z-YVAD-FMK (BioVision Int, Brussels, Belgium) (50 µM) affects IL-1β production were carried out by applying it 45 min before and throughout the phagocytosis assay.

### Measurement of IL-1β and ATP

LPS primed macrophages were co-incubated with appropriate target cells and after the 2 h of co-incubation supernatants were collected and IL-1β was measured by using ELISA (R&D DuoSet, Budapest, Hungary). In experiments in which CASPASE-1/NALP3 knockout mice macrophages were used, immunoreactive levels of IL-1β were measured in conditioned medium by using a Milliplex mouse cytokine kit (MPXMCYTO-70K-01, Merck Millipore, Overijse, Belgium) according to the manufacturer’s instructions and analyzed on a Bio-Plex 200 (Bio-Rad, Nazareth Eke, Belgium).

The concentration of ATP was measured in supernatants by using ATPliteTM Luminescence Assay System (Perkin Elmer, Budapest, Hungary) according to the manufacturer’s instructions, and light production was measured on a VICTOR2TM (Perkin Elmer) reader.

### Intraperitoneal Injection of Dying/dead Ba/F3 Cells and Phenotyping of Peritoneal Exudate Cells

Autophagy, apoptosis and necrosis were induced in Ba/F3 cells as described above. Dying autophagic, apoptotic or necrotic cells and live cells were harvested by centrifugation, washed three times with sterile D-PBS (Invitrogen) and resuspended in D-PBS at a density of 40×10^6^ cells/ml. Balb/c mice syngeneic for Ba/F3 cells (8–10 weeks old, Janvier, Bio Services BV, The Netherlands, n = 4-5 mice per group) were intraperitoneally injected with 10×10^6^ cells/mouse in 0.250 ml of D-PBS. Equal volumes of D-PBS were injected as negative controls. Sixteen hours after injection, animals were euthanized by CO_2_ exposure, and peritoneal exudate cells (PECs) were isolated by peritoneal lavage. The red blood cells were lysed with ACK cell lysis buffer (Lonza Walkersville, Basel, Switzerland). The number of PECs was counted in a hemocytometer using trypan blue and phenotyped by flow cytometry. All experimental procedures were approved by the local Ethics Committee of Ghent University–VIB.

### Flow Cytometric Analysis of PECs Phenotypes

PECs (5×10^5^) were incubated with rat anti-mouse antibody 2.4G2 (BD Pharmingen, Erembodegem, Belgium) for 30 min at 4°C to block FcγRIIB/III receptors. Since apoptotic cells were treated with doxorubicin, which has a broad range of auto-fluorescence, we divided each sample and used two different stainings in order to identify monocytes, macrophages, eosinophils and neutrophils. To quantify monocytes, macrophages, neutrophils and eosinophils, the PECs were stained with anti-mouse antibodies F4/80-APC (clone BM8, eBioscience) and CD11b-APC-Cy7 (clone M1/70, BD Pharmingen). To identify neutrophils, the PECs were stained with anti-mouse antibodies Ly-6G-APC (clone 1A8, BD Pharmingen) and CD11b-APC-Cy7 (clone M1/70, BD Pharmingen). All the stainings were done for 30 min at 4°C in PBS. Just before flow cytometric analysis on BD LSR-II (BD Biosciences), 1.25 nM of Sytox Blue dead cell stain was added (Invitrogen) to exclude dead cells from the measurements. Data were acquired and analyzed by BD FACSDiva software (BD Biosciences). The following cell populations were discriminated: macrophages (F4/80^high^ CD11b^high^), monocytes (F4/80^medium^ CD11b^high^), eosinophils (F4/80^medium^ CD11b^medium^) and neutrophils (CD11b^+^ Ly6G^high^). To determine the number of cells in each specific cell population, the total number of PECs was multiplied by the percentage of specific cell population mentioned above.

### Statistical Methods

Results are expressed as the mean ± SEM for the number of assays indicated. For multiple comparisons of groups statistical significance was calculated and evaluated by one-way ANOVA followed by Tukey post-hoc test. In comparison of two groups non-bias two-tailed unpaired student t test was used.
